# Empirical codon substitution matrix

**DOI:** 10.1186/1471-2105-6-134

**Published:** 2005-06-01

**Authors:** Adrian Schneider, Gina M Cannarozzi, Gaston H Gonnet

**Affiliations:** 1 Institute of Computational Science, Swiss Federal Institute of Technology, Zurich, Switzerland

## Abstract

**Background:**

Codon substitution probabilities are used in many types of molecular evolution studies such as determining Ka/Ks ratios, creating ancestral DNA sequences or aligning coding DNA. Until the recent dramatic increase in genomic data enabled construction of empirical matrices, researchers relied on parameterized models of codon evolution. Here we present the first empirical codon substitution matrix entirely built from alignments of coding sequences from vertebrate DNA and thus provide an alternative to parameterized models of codon evolution.

**Results:**

A set of 17,502 alignments of orthologous sequences from five vertebrate genomes yielded 8.3 million aligned codons from which the number of substitutions between codons were counted. From this data, both a probability matrix and a matrix of similarity scores were computed. They are 64 × 64 matrices describing the substitutions between all codons. Substitutions from sense codons to stop codons are not considered, resulting in block diagonal matrices consisting of 61 × 61 entries for the sense codons and 3 × 3 entries for the stop codons.

**Conclusion:**

The amount of genomic data currently available allowed for the construction of an empirical codon substitution matrix. However, more sequence data is still needed to construct matrices from different subsets of DNA, specific to kingdoms, evolutionary distance or different amount of synonymous change. Codon mutation matrices have advantages for alignments up to medium evolutionary distances and for usages that require DNA such as ancestral reconstruction of DNA sequences and the calculation of Ka/Ks ratios.

## Background

Models for codon substitutions are used in computational biology for a wide range of applications such as reconstructing ancestral DNA sequences, determining Ka/Ks ratios to identify periods of adaptive evolution and aligning coding DNA.

Methods for estimating mutation matrices from observed substitutions in sequence alignments of proteins were established by Dayhoff [1]. These matrices contain the probabilities of amino acid mutations for a given period of evolution and have long been used for scoring protein sequence alignments, evolutionary studies and homology searches.

More than a decade ago, when large-scale protein databases became established, several amino acid substitution matrices based on observed mutation counts in protein alignments were constructed [2-4], replacing the original Dayhoff matrices that were based on relatively few alignments.

However, to describe substitutions at the codon level, parameterized models have been developed [5,6] and are widely used in the study of molecular evolution.

In the same way that the growth of protein databases allowed refined construction of amino acid substitution matrices, the recent increase of nucleotide sequence data made it possible to apply these methods at the codon level. The matrices presented here were constructed using the approach of Gonnet [4]. This implies that pairwise alignments using full dynamic programming [7,8] were used in order to count the observed transitions between codons. The sequence data was taken from the complete vertebrate genome databases of *ENSEMBL *[9].

## Results

The additional files contain the 64 × 64 matrices presented here. Various aspects of the matrices will be discussed in this section. We present the matrix with the exact counts of the observed substitutions (Additional file 1), the matrix containing the substitution probabilities derived therefrom (Additional file 2) and a matrix containing similarity scores for all possible substitutions (Additional file 3).

### Substitution counts

The 17,502 alignments that have been used to construct the matrix presented here contained 8.3 million aligned codon pairs. From each of these aligned pairs, the number of each of the 3730 (61 × 61 + 3 × 3) possible substitutions were counted. The difference between the numbers of frequent and rare substitution is high with the most frequent substitution (the GAG identity) being observed 153,040 times, and the rarest substitution, between TGG and GAG, being counted only 45 times, about 3400 times less often.

To estimate the precision of the count of the rarest substitution, a binomial distribution of the counts can be assumed. The substitution with minimal count *c*_*min *_occurs with probability *p *= 45/(8.3·10^6^) = 5.4·10^-6^. The variance of a binomial distribution is *σ*^2 ^= *N*(1 - *p*)*p *and thus for very small *p*, the variance of *c*_*min *_is almost equal to *c*_*min *_= 45 and the standard deviation *σ *is 6.7.

This means that although a very large amount of data is used to construct the matrix, it is just enough to produce codon counts that are of a tolerable accuracy for rare transitions. Only a further increase of high-quality genomic data will allow the clustering of the data into specific subsets. These possibilities will be discussed below.

### Substitution probabilities

The mutation matrix *M *constructed from the counts contains the substitution probabilities for the individual codons. Entry *M*_*i*,*j *_gives the probability that codon *j *mutates to codon *i*. (As a consequence, each column of *M *sums to 1).

A convenient measure to express the amount of mutations in a matrix is the percentage identity



with *f*_*i *_being the natural frequencies of the codons. For the matrix reported here, *p *is .35, meaning that in the alignments used, 65% of the codons have undergone substitution (to any other codon, thereby involving up to three nucleotide changes).

It is also possible to calculate the percentage of identical amino acids resulting from this matrix:



In the second sum, *i *goes over all codons that code for the same amino acid as *j *does. The result for *p*_*AA *_is .69, therefore 31% of the amino acids are expected to mutate. This allows the determination of the relationship between the codon substitution matrices and amino acid substitution matrices, because the amino acid PAM distance can be derived from the percentage of amino acid identity.

Analogously to the definition of 1 PAM, 1 Codon-PAM can be defined as the distance at which 1% of the codons undergo substitution. Again, a codon substitution can involve up to three nucleotide base changes. The substitution matrix for any distance *d *is approximated by raising the 1 CodonPAM matrix to the power of *d*.

The relationship between CodonPAM, PAM and *f*2 is shown in Figure 2. It shows that amino acid PAM increases almost linearly with CodonPAM. The curve is slightly steeper for the low distances and flattens with increasing distance. The amount of synonymous substitution decays from 1 to .51 (f2 being a measure of synonymous mutation described in the Methods section).

### Mutation scores

Since the substitution probabilities are influenced by the codon frequencies, it is not possible to see directly which substitutions occur more than expected and which occur less. This issue is corrected in the scoring matrix *D*, where entry *D*_*i*,*j *_expresses how much more likely it is that codons *i *and *j *were derived from a common ancestral codon compared to a random pairing of them. A higher score for a transition means that this transition is indeed more likely than one with a lower score. The scoring matrix is symmetric, i.e. the transition from codon *i *to *j *has the same score as the transition from *j *to *i*.

Table 1 displays average scores for different categories of substitutions. It confirms the fact that synonymous substitution scores are generally higher than non-synonymous scores. But it can also be seen that as more nucleotides change, the scores become lower. Synonymous substitutions in which all three bases change, have lower scores than non-synonymous substitutions with only one base change.

## Discussion

### Synonymous mutations

It has been observed that different genes have different Ka/Ks ratios and therefore the fraction of synonymous substitutions will differ between different gene pairs having a certain PAM distance. This is because there are no strong selective constraints on synonymous substitutions and therefore the number of these substitutions accumulates in a clock-like manner [10] while the number of nonsynonymous substitutions is governed by functional constraints.

**Figure 1:**
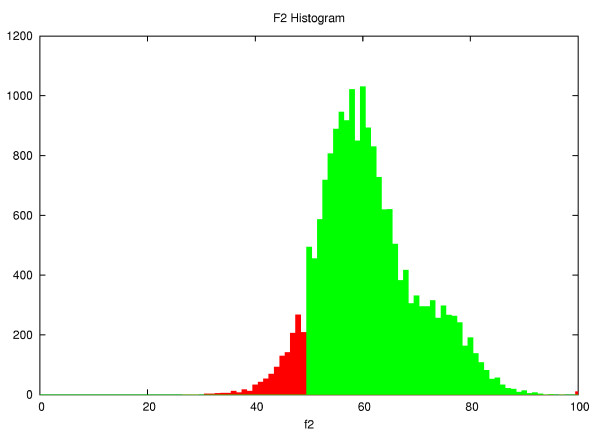
**f2 histogram. **Histogram of the f2 values from the 17,502 alignments used to construct the matrix.

**Figure 2:**
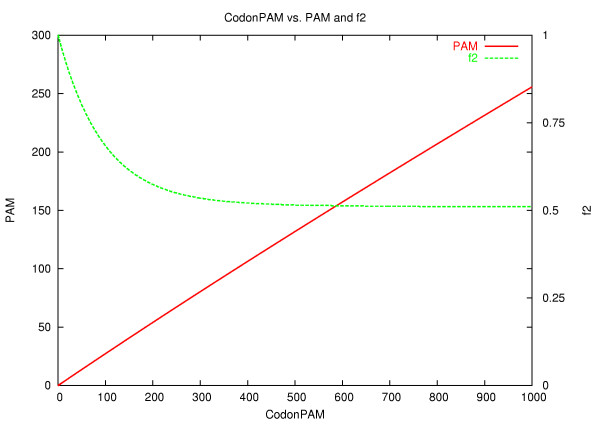
CodonPAM vs PAM and f2

Increasing amounts of genomic data would allow the construction and comparison of matrices from alignments with differing amounts of synonymous and non-synonymous substitutions, representing a two-dimensional array of matrices, where one dimension is the evolutionary distance and the other corresponds to the amount of synonymous change. Unfortunately, the current size of the nucleotide databases does not yet allow such a clustering of the available data. Instead, the alignments selected to construct the matrices were filtered to fall within a window of synonymous mutations, thereby excluding the most extreme values. (see the Methods section for details). Figure 1 shows the distribution of the alignments' f2 values.

### Range of applicability

One possible application of scoring matrices is protein and coding DNA alignment. In order to compare alignments based on amino acid substitution matrices and the codon matrices presented here, the likelihood scores are compared. Since these scores express the probability ratios of the two sequences having evolved from a common ancestor to them being aligned by random chance, they serve as a confidence measure of an alignment. The higher the score, the higher the likelihood that the alignment is by reasons of ancestry than by random chance.

As the likelihood scores serve as an indicator of alignment quality, orthologous sequences for species pairs of various distance and classes were used to determine when codon matrix based alignments produced higher scores than amino-acid PAM matrices.

Table 2 displays for several pairs of species, the number of orthologs used to perform the alignment analysis, the average PAM distance between these orthologs (found by selecting the highest-scoring PAM matrix) and the average ratio of codon based scores to amino acid based scores. A number greater than 1 means that on an average, the codon based scores were higher.

The result is that for closely related species, the codon based scores are always higher, but the more distant two species are, the better the performance of the amino acid based alignments. An interesting point is that although codon mutations in different sets of species were found to be significantly different (χ^2 ^tests, data not shown here), the above finding holds not only for the vertebrates, from which the matrices were constructed, but also for the invertebrates, yeasts and even bacteria.

From the results in Table 2, a PAM distance smaller than 50 would favor the use of codon substitution matrices instead of amino acid based matrices.

## Conclusion

Because codon substitution matrices are substantially bigger than amino acid matrices and also because some of the substitutions are extremely rare compared to the most frequent ones, large amounts of genomic data are necessary to model the transitions accurately. The 17,502 alignments used here produce enough aligned codons to fulfill this criterium, but do not allow further clustering of the data set in order to create more specific matrices.

The codon substitution matrix presented here is to our knowledge the first based entirely on empirical data and can serve in many fields of computational biology. We have found that at long distances, when the synonymous mutations have reached saturation, amino acid matrices are better suited for alignments and long-distance homology searching. Codon mutation matrices have advantages for alignment up to medium evolutionary distances and for usages that require DNA such as ancestral reconstruction of DNA sequences and the calculation of Ka/Ks ratios.

## Methods

The basic methods to create scoring matrices are well established. The construction of codon substitution matrices is analogous to that of amino acid transition matrices. The main difference lies in the fact that codon matrices are much larger (4096 elements (64 × 64) instead of 400 elements (20 × 20)). In addition, the stop codons need special consideration. Substitutions between stop codons and sense codons are assumed to be very rare because the effect of such substitutions on the function of the protein would probably be very serious thus the chance of acceptance is very small and usually limited to the 3' end of the nucleotide sequence. This makes it almost impossible to observe such events. Therefore, these substitutions are not included in the matrices presented here. Substitutions between stop codons, however, are counted and thus also contained in the matrices. This means that the 64 × 64 matrices are block diagonal composed of a 61 × 61 matrix for the coding codons and a 3 × 3 matrix describing substitutions between stop codons.

### Using orthologs

The matrices are constructed from pairwise alignments of orthologous sequences from five vertebrates – human (*Homo sapiens*), mouse (*Mus musculus*), chicken (*Gallus gallus*), frog (*Xenopus tropicalis*) and zebrafish (*Brachydanio rerio*). The complete genome databases from *ENSEMBL *[9] were used for this purpose. Using only orthologs has the advantage that no gene is overrepresented in the data set. This is because a particular gene can have many paralogous genes in a genome, but we allowed at most one ortholog per other genome.

### Circular tours

When counting the substitutions in alignments from all pairs of species, substitutions that occurred early in the tree are counted more often than those that happened later, because paths between two species include the branches near the root more often than those near the leaves. This bias can be prevented by using only species pairs along a circular tour. This way every branch of the tree (and therefore every substitution that ever happened in the history of the genes) examined, is counted at most twice. Concretely, this means that only the orthologs between human and mouse (3107 pairs), mouse and chicken (3691 pairs), chicken and frog (3671 pairs), frog and fish (3441 pairs) and fish and human (3592 pairs) are counted, resulting in 17,502 alignments.

### Counting substitutions

These alignments must fulfill several criteria: 1) they should all be of similar evolutionary distance because substitution probability depends on evolutionary distance. A trade-off exists for the acceptable range of distances as including a broad range of distances, increases the amount of data but at the same time blurs the distance specific information. 2) The alignments must be of a distance that is high enough to allow the observation of rarer substitutions. 3) There must be enough alignments to have statistically significant data for the rare substitutions. After some observations performed on a subset of the data, a distance range of 25 to 60 PAM (57% to 78% identity) of the protein alignments was found to best satisfy these criteria.

Another selection criterium was based on the amount of synonymous substitutions between the sequences to eliminate saturation effects from the observed synonymous substitutions. One way to estimate the amount of synonymous substitution is *f*2, the percentage of conserved synonymous codons at two-fold redundant amino acid sites. Two identical sequences have an *f*2 value of 1 and it will decay to a value near .5 for increasing amounts of substitutions. An *f*2 range between .50 and .95 was found to exclude the most extreme cases of synonymous substitutions while leaving enough alignments to fill the matrices.

Figure 1 shows the distribution of *f*2 values for all alignments within the PAM range of 25 to 60. The sequence pairs with *f*2 values between .50 and .95 are shown in green and were used to construct the matrices, while the alignments corresponding to the red bins were discarded.

Full dynamic programming [4,7,8] was employed in order to construct the alignments. The DNA alignment was obtained by mapping the coding DNA to the aligned proteins. Directly aligning the DNA was not yet possible, because no a priori knowledge about codon similarities was assumed. However, in the refinement steps (see further below), the DNA itself was aligned using the codon substitution matrices from the previous refinement round.

Once the sequences were aligned, the actual substitution matrices could be computed. This included counting the observed codon substitutions in the collected alignments and storing them in a 64 × 64 count matrix *C*. Since the direction of a substitution is not known, for each observed substitution between codons *i *and *j*, *C*_*i*,*j *_as well as *C*_*j*,*i *_is increased by 1/2. Insertion or deletion sites were ignored since they provide no information about actual substitutions. From the count matrix, the mutation matrix was derived according to equation 3:



#### Calculating similarity scores

The transition scores for a given substitution matrix express the relative probabilities of two codons originating from a common ancestor compared to the probability of them being paired by random chance. The logarithm is taken to make the scores additive, thereby speeding up the computation of alignment scores. *D *is calculated as



where *f*_*i *_is the frequency of codon *i *in the observed data set. The factor 10 is used for purely historical reasons.

In an iterative process, the sequence pairs were aligned again with dynamic programming [7,8] but this time directly on the codon sequences using the substitution matrices obtained before. Exponentiation of the mutation matrix was used to approximate matrices for different evolutionary distances [12], allowing a maximum-likelihood estimation of the best-fitting matrix to align the sequences. From these new alignments new mutation matrices and finally scoring matrices were constructed in the way described above, until after six iterations a sufficient convergence of the matrix was reached.

## Authors' contributions

GMC initiated and guided this project and contributed to the programming. AS did the majority of the programming and analysis. Some of the work was based on previous research by GHG. All work was supervised by GHG. AS and GMC drafted the manuscript which was approved by GHG.

## Supplementary Material

Additional File 1 The 64 × 64 matrix containing the observed substitution counts as described in the results section. Click here for file

Additional File 2 The 64 × 64 matrix containing the substitution probabilities. Click here for file

Additional File 3 The 64 × 64 matrix of the transition scores. Click here for file

## Figures and Tables

**Table 1:** Analysis of the scores. Average scores for different categories of substitutions. The stop codons are excluded from this analysis.

	n Substitutions	Avg. Score
Identity	61	12.9

Synonymous: all	87	8.7
Synonymous: 1 base change	67	10.0
Synonymous: 2 base changes	14	6.6
Synonymous: 3 base changes	6	-1.7

Non-syn.: all	1743	-7.3
Non-syn.: 1 base change	196	-1.3
Non-syn.: 2 base changes	770	-5.9
Non-syn.: 3 base changes	777	-10.3

All Substitutions	1891	-5.9

**Table 2:** Range of applicability. Ratios of likelihood scores for amino acid and codon based alignments for orthologs between several species pairs, where *N *is the number of orthologs used.

	N	Avg. PAM	Scores ratio
*Homo sapiens*			
vs. *Mus musculus*	14655	17.4	1.150
vs. *Gallus gallus*	9272	29.3	1.060
vs. *X. tropicalis*	9953	39.1	1.026
vs. *B. rerio*	7507	43.7	1.013

*Drosophila melanogaster*			
vs. *A. gambiae*	5059	57.3	.995
vs. *H. sapiens*	3371	77.5	.959
vs. *C. elegans*	2156	88.8	.945

*Saccharomyces cerevisiae*			
vs. *C. glabrata*	3467	52.7	1.002
vs. *A. gossypii*	2909	61.4	.978
vs. *H. sapiens*	1187	94.1	.931

*Escherichia coli*			
vs. *E. coli *strain O6	3156	2.0	1.323
vs. *Salmonella typhi*	2557	14.2	1.067
vs. *P. aeruginosa*	1234	71.6	.980
vs. *B. japonicum*	765	90.2	.959
